# Substrate Integrated Waveguide (SIW)-Based Wireless Temperature Sensor for Harsh Environments

**DOI:** 10.3390/s18051406

**Published:** 2018-05-03

**Authors:** Qiulin Tan, Yanjie Guo, Lei Zhang, Fei Lu, Helei Dong, Jijun Xiong

**Affiliations:** 1 Key Laboratory of Instrumentation Science & Dynamic Measurement, Ministry of Education, North University of China, Taiyuan 030051, China; 18235140097@163.com (Y.G.); 18734136023@163.com (L.Z.); lufei_55@163.com (F.L.); donghelei@nuc.edu.cn (H.D.); xiongjijun@nuc.edu.cn (J.X.); 2 Science and Technology on Electronic Test and Measurement Laboratory, North University of China, Taiyuan 030051, China

**Keywords:** substrate integrated waveguide, wireless temperature sensor, harsh environment, resonant frequency, CPW-fed antenna

## Abstract

This paper presents a new wireless sensor structure based on a substrate integrated circular waveguide (SICW) for the temperature test in harsh environments. The sensor substrate material is 99% alumina ceramic, and the SICW structure is composed of upper and lower metal plates and a series of metal cylindrical sidewall vias. A rectangular aperture antenna integrated on the surface of the SICW resonator is used for electromagnetic wave transmission between the sensor and the external antenna. The resonant frequency of the temperature sensor decreases when the temperature increases, because the relative permittivity of the alumina ceramic increases with temperature. The temperature sensor presented in this paper was tested four times at a range of 30–1200 °C, and a broad band coplanar waveguide (CPW)-fed antenna was used as an interrogation antenna during the test process. The resonant frequency changed from 2.371 to 2.141 GHz as the temperature varied from 30 to 1200 °C, leading to a sensitivity of 0.197 MHz/°C. The quality factor of the sensor changed from 3444.6 to 35.028 when the temperature varied from 30 to 1000 °C.

## 1. Introduction

As a basic physical parameter, temperature plays an important role in the aerospace and automotive industries, mine operations and other industries. Temperature monitoring in harsh environments has attracted a large number of researchers to explore it in depth, because an appropriate environment temperature can achieve higher energy efficiency and apply a higher safety factor. Many kinds of temperature sensors have been developed, thus far.

The most common temperature sensors include optical fiber temperature sensor [1,2,3], thermocouple, surface acoustic wave (SAW) temperature sensor [4,5], inductive capacitive (LC)-based resonant temperature sensor [6,7], microwave-based scattering temperature sensor [8,9], etc. For the optical fiber-type temperature sensors, the main attractive features are miniaturization, immunity to electromagnetic radiation, high stability, and extraordinary resistance to corrosive environments; however the requirement of high fabrication precision makes it difficult to be popularize [1,2,3]. For the SAW temperature sensors, the measured signal can be easily contaminated, and the chemical instability of the substrate material severely limits the test range of the sensor [4,5]. Nowadays, new piezoelectric materials are available, e.g., La_3_Ga_5_SiO_14_. They have no phase change in the temperature range from room temperature to the melting point; however, these piezoelectric materials are extremely expensive. In [6], a temperature sensor based on an inductive capacitive (LC) resonance circuit has been successfully tested at 700 °C, using low temperature co-firing ceramic (LTCC). Moreover, in [7], a temperature sensor based on an LC resonance circuit has been successfully tested at 900 °C, which was fabricated based on high-temperature co-firing ceramic (HTCC). However, this type of sensor cannot be used near metal surfaces because the magnetic field will be absorbed by the metal surface. In addition, these sensors have low quality factor. In [8], a wireless passive dielectrically loaded resonator temperature sensor was presented, which has been successfully tested at 800 °C. In [9], two dielectric resonance temperature sensors based on Si_6_B_1_ and Si_4_B_1_ ceramics are measured up to 1050 °C and 1300 °C, respectively, sensors structure in these two literatures are very similar, and the thickness of these sensors are about five millimeters, which makes it less convenient to install inside an engine. 

The thickness of the substrate integrated waveguide (SIW) is thinner than these structures. It can be easily integrated with other planar circuits and has a high-quality factor [10,11,12]. In addition, the substrate integrated waveguide also has a good performance for transmitting electromagnetic waves. At present, the substrate integrated waveguide is widely used in filters, antennas, directional couplers, and wavelength devices; it is also an important part of the resonator [13]. However, there are still few studies on RF-based sensors using SIW, only few sensors have been reported to detect gas concentration, humidity and fluid composition, etc. In [14], an SIW resonator was integrated with a micro-strip line to prepare a gas sensor for hydrogen detection. In [15], the resonant technique was applied to the analysis of an SIW sensor for humidity detection. In [16], a novel microfluidic-integrated microwave sensor was designed based on the resonance method and implemented by using an SIW structure combining with a rectangular slot-antenna, and which has shown some of its potential to accurate quantification of liquid mixtures and further liquid characterization through measurement of the relative permittivity. In [17], a novel SIW resonator sensor with high-unloaded quality factor was designed for fast and reasonably accurate complex permittivity measurements. The resonant characteristics of the sensor are influenced by liquids through a slot opened on the top plane. In [18], a Microfluidic sensor based on the SIW structure was presented to achieve a real time characterization of fluids across the lower Ultra-wideband frequency band. In [19], a high-Q and miniaturized complementary split ring resonator (CSRR)-loaded substrate integrated waveguide (SIW) microwave sensor for the detection of cracks in metallic materials was presented.

In this paper, a wireless passive resonator based on the substrate integrated waveguide (SIW) structure is presented. The SIW sensor is used for temperature detection. The relative permittivity of the substrate material changes with the temperature [20], correspondingly the sensor resonant frequency varies monotonically with the temperature. The sign of the frequency shift of the temperature sensor is tested by a network analyzer, and the quality factor is also recorded during the test.

This paper is organized as follows. Section 2 presents the principle of the SIW temperature sensor. Section 3 describes the parameters and the sensor preparation. The measurements of the fabricated sensor are presented in Section 4. The performance of the temperature sensor is analyzed in Section 5 and a conclusion is given in Section 6.

## 2. Working Principle of the Temperature Sensor

**Figure 1:**
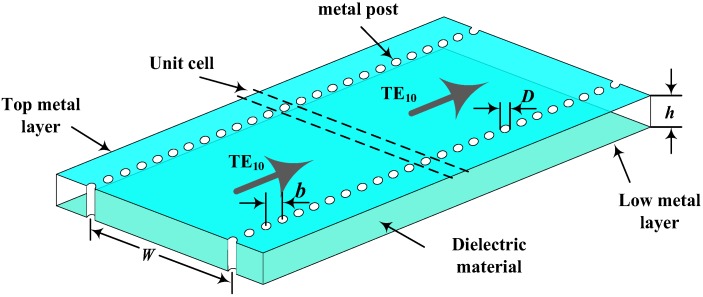
Geometry of a substrate integrated waveguide. TE: transverse-electric wave mode.

The traditional substrate integrated waveguide (SIW) structure is composed of upper and lower metal plates along with a two-row metal through-hole cylinder of the substrate material. The cavity is filled with media material as shown in Figure 1. The transmission performance of the electromagnetic wave in an SIW is similar to that in rectangular waveguide structures. The upper and lower metal surfaces of the dielectric substrate can be regarded as the upper and lower waveguide walls of the corresponding rectangular waveguide. The two rows of the metal through-hole cylinders constitute the two metal sidewalls of the conventional rectangular metal waveguide which limit the external radiation of the electromagnetic waves. The geometry of the traditional substrate integrated waveguide is shown in Figure 1.

Because of the discontinuity of the metal sidewall through-hole cylinder of the SIW, the transverse-magnetic (TM) mode wave cannot form a stable current through the metal sidewall through-hole; thus, the TM mode wave cannot be transmitted efficiently in the SIW. In contrast, the transverse-electric (TE) mode of the wave can form a stable current through the sidewall; therefore, only the TE mode wave can be propagated in the SIW. In addition, the gap between the metal through-hole in the SIW sidewalls causes the electromagnetic wave to leak during the propagation.

In [21,22], the authors concluded that in order to reduce the electromagnetic leakage in the SIW, its parameter size should meet the following conditions:

(1) $$D<0.1\lambda_{g},\;b<4D,\;D<0.2W_{eff}$$

where *D* represents the diameter of the sidewall metalized through-hole cylinder; *b* represents the center-to-center spacing of two adjacent metalized through-hole cylinders in the same horizontal section, *λ_g_* represents the effective wavelength of the guided wave along the cylindrical wall of the SIW, and *W_eff_* represents the effective width of the equivalent rectangular waveguide.

The temperature sensor presented in this paper is composed of a substrate integrated circular waveguide (SICW) resonator and an aperture antenna. For the SICW, *R_eff_* replaces the *W_eff_* parameter of the traditional SIW structure; it represents the equivalent radius of the SICW resonator. The relationship between the effective radius and the actual radius of the (SICW) resonator can be determined by Equation (2) [23].

(2) $$R_{eff}=R-\frac{D^{2}}{0.95b}$$

**Figure 2:**
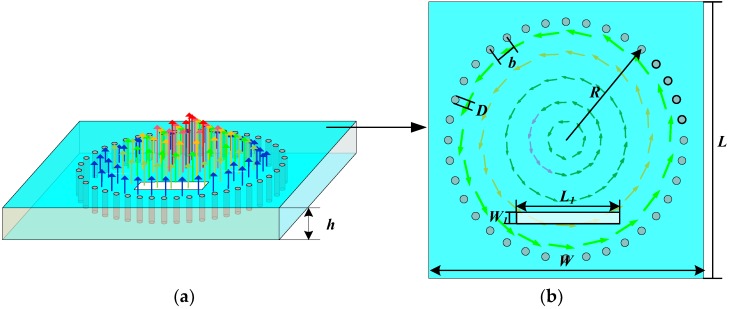
The structure and electromagnetic field distribution (Eigen-modes) of the substrate integrated waveguide (SIW) temperature sensor: (**a**) Electric field distribution (**b**) Magnetic field distribution.

The actual radius *R* of the SICW resonator represents the distance between the center of any of the metalized through-holes in the same horizontal section and the geometric center of the SICW resonator. As shown in Figure 2, this new temperature sensor is based on the SICW resonator, and the proposed structure is designed on a sheet of alumina ceramic substrate with a dielectric constant *ε_r_* of 9.8 (at room temperature). The resonant frequency of the SICW can be expressed by Equation (3) [24]. The electromagnetic field distribution (Eigen-modes) of the sensor is shown in Figure 2.

(3) $$f_{r}=\frac{c}{\sqrt{\mu_{r}\varepsilon_{r}}}\cdot \frac{P_{11}}{2\pi R_{eff}}$$

where *c* represents the speed of light in vacuum, *P*_11_ represents the first zero-point of the First-order Bessel function, which equals 2.4048; *μ_r_* represents the permeability of the dielectric substrate material; and *ε_r_* represents the relative permittivity of the dielectric material. The top view of the temperature sensor is shown in Figure 1b. Through Equations (2) and (3), it can be concluded that the resonant frequency of the sensor is affected by the relative permittivity of the dielectric material. The dielectric constant of the alumina ceramic material increases with the increasing temperature [25,26], causing the resonant frequency of the temperature sensor to decrease.

## 3. Design and Fabrication of the Temperature Sensor

In this study, we set the resonant frequency of the sensor at around 2.4 GHz. The parameter size of the SIW resonator can be calculated according to Equations (1)–(3). The initial dimensions parameters of the SIW calculated by Equations (2) and (3) are shown in Table 1, where *L* and *W* represent the length and width of the substrate. 

The thickness of the substrate material is substantially unrelated to the resonant frequency of the SIW sensor, while it has a great impact on the quality factor. The High Frequency Structure Simulator (HFSS) was used to determine the thickness of the SIW. The resonant frequency and the quality of the sensor can be obtained through the Eigen-mode simulation in the HFSS. When the thickness of the SIW varies from 0.7 to 1.3 mm, the corresponding changes of the resonant frequency and the quality factor are shown in Table 2.

The simulation results show that the sensor resonant frequency has almost no change when the thickness changes from 0.7 to 1.3 mm, while the quality factor increases as the thickness of the substrate material increases. These results are consistent with the conclusions mentioned above. However, when the substrate thickness exceeds one millimeter, it becomes more difficult to machine the sidewall through-hole cylinder in the substrate material; thus, we chose the alumina ceramic *h* = 1.0 mm as the substrate material of the sensor. A rectangular aperture was integrated onto the surface of the SIW resonator; it works as a response antenna to couple the electromagnetic signal into the SICW resonator. Then, the HFSS drive-mode simulation was used to determine the location and size of the aperture antenna. 

**Figure 3:**
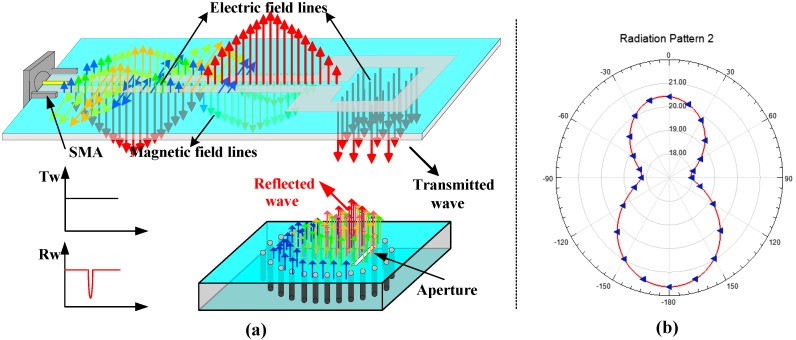
(**a**) The schematic of the temperature sensor signal transmission mechanism; (**b**) The near-field radiation pattern of the slot antenna along the direction of the electric field.

**Figure 4:**
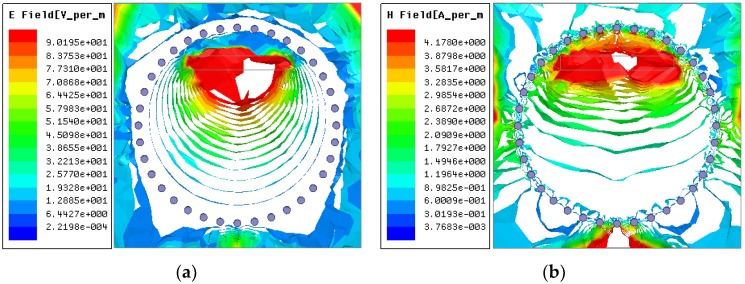
The electromagnetic field distribution cloud of the SIW temperature sensor excited by the coplanar waveguide (CPW) antenna: (**a**) Electric field distribution; (**b**) Magnetic field distribution.

A broad band coplanar waveguide (CPW)-fed square-slot antenna connected with a SMA connector was used as an excitation source during the simulation process. The parameters of the CPW-fed antenna used in the HFSS simulation were presented in [27], and the electromagnetic field distribution of the antenna is shown in Figure 3. By comparing the electromagnetic-field distribution of the sensor (Eigen-modes), it can be concluded that when the relative position between the CPW-fed antenna and the sensor is as shown in Figure 3a, the sensor produces the best resonance performance under the electromagnetic excitation of the antenna, the near-field radiation pattern of the slot antenna along the direction of the electric field line as shown in Figure 3b. The electromagnetic-field distribution inside the sensor is shown as Figure 4.

**Figure 5:**
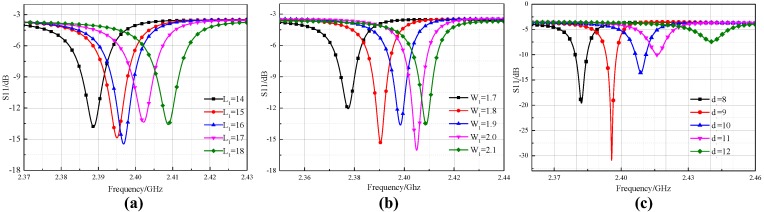
The result of the parameters sweep. (**a**) *L*_1_ varies from 14 to 18 mm; (**b**) *W*_1_ varies from 1.7 to 2.1 mm; (**c**) *d* varies from 8 to 12 mm.

For the aperture antenna, the three primary parameters—aperture length *L*_1_, width *W*_1_, and distance *d* between the aperture and SICW resonator center—determine the impedance matching between the aperture and the resonator; thus, affecting the coupling effect between the sensor and the interrogation antenna. The sweep result of these parameters is shown in Figure 5. A sharper and lower negative peak indicates a good match between the antenna and the sensor. Hence, *L*_1_ = 16 mm, *W*_1_ = 2.0 mm and *d* = 9 mm were determined as the final dimension.

**Figure 6:**
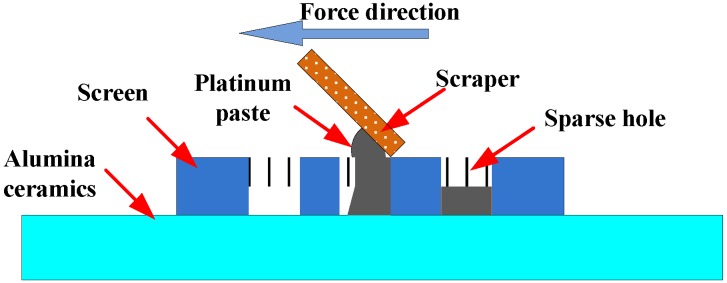
Screen printing process diagram.

After all the parameters were determined, laser-drilling technology was used to fabricate the sidewall through-hole cylinders (*D* = 1 mm) on the alumina substrate material (*h* = 1 mm). The number of sidewall through-hole cylinders was 36. Screen-printing technology with ESL5541A platinum paste was used to prepare the lower metal plate and integrate the aperture antenna on the upper surface of the SIW. The screen-printing process diagram is shown in Figure 6.

The screen-printing technology is used in this study for its convenient operation. In comparison with metal-sputtering technology, the thickness of the metal layer is higher, the conductivity is better, and the metal layer does not easily fall off the alumina substrate in a high-temperature environment. The most important point is that the metal layer formed by sputtering contains nano-scale particles and cannot withstand extremely high temperatures, while the metal layer obtained by screen-printing is robust in harsh environment with high temperatures.

**Figure 7:**
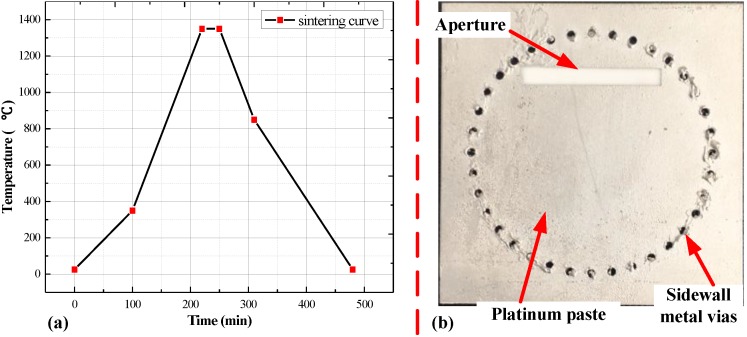
The sintering curve of the platinum paste and the fabricated sensor (**a**) the sintering curve of ESL5541A platinum paste; (**b**) fabricated temperature sensor.

To fill the sidewall cylinder, ESL5541A platinum paste was used as the primer in the sidewall through-hole cylinder, then the structure was heated at 100 °C for 20 min. This operation was repeated several times to ensure a sufficient amount of platinum paste inside the sidewall cylinder. Finally, the sensor was placed in the furnace for sintering with the maximum temperature of 1350 °C, because the maximum sintering temperature of ESL5541A platinum paste is 1350 °C. The sintering curve of the platinum paste and the fabricated sensor are shown in Figure 7.

## 4. Temperature Measurements

**Figure 8:**
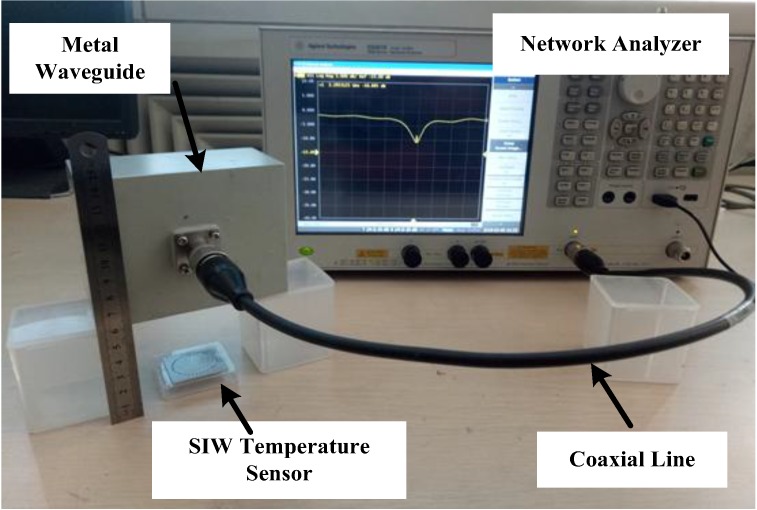
The test photo when using a metal waveguide at room temperature.

In order to study the influence of the test distance on the sensor signal, the CPW-fed square-slot antenna and the metal waveguide were used to test the sensor at room temperature, respectively. Through testing, it has been found that when using a metal waveguide to passively feed the sensor, the measured frequency signal is better, and we can see that when adjust the sensing distance to about six centimeters, we can still detect valid sensor signals, as shown in Figure 8. However, metal waveguides are not suitable for use in high temperature test experiments.

**Figure 9:**
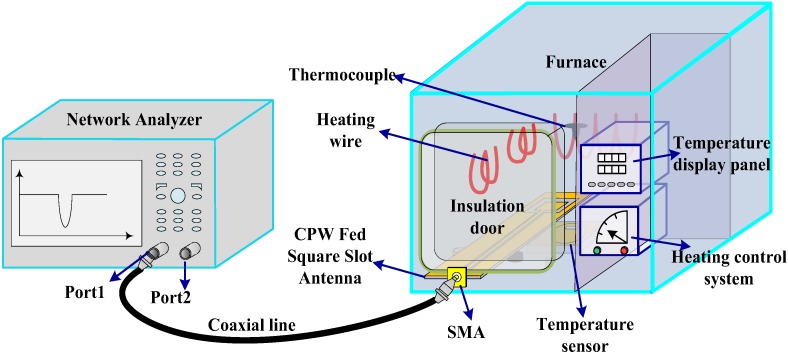
The schematic diagram of the temperature test platform.

To measure the response of the prepared sensor in a high-temperature environment, several wireless test platforms were built. The schematic diagram of the test platform is shown in Figure 9. The Nabertherm LHT 02/16 muffle furnace was chosen to provide high temperature conditions for testing. It has a built-in thermocouple sensor to achieve real-time temperature monitoring. The built-in control micro-system on the furnace allows the furnace to set the multistage heating curve. During the test, the temperature sensor was placed at the center of the furnace. A CPW-fed square slot antenna that connected to a coaxial line was used to transmit electromagnetic-wave signals to the sensor, and also act as an integrated antenna to receive the reflected electromagnetic signals emitted by the temperature sensor. An insulated door was used to protect the SMA of the CPW antenna from damage caused by the high temperature environment. One end of the coaxial line was connected with the CPW antenna, and the other end was connected to the network analyzer E5061-b, so the echo scattering signal could be displayed in real time on the network analyzer’s display panel.

During the test process, it should be noted that the relative position of the interrogation antenna and the aperture has a significant impact on the readout signal due to the directivity and polarization direction of the interrogation antenna and aperture. When the aperture on the surface of the sensor is parallel to the short side of the CPW-fed square-slot antenna, the coupling effect is optimal, and the maximum sensor signal strength could be obtained. This is consistent with the results obtained from the simulation.

**Figure 10:**
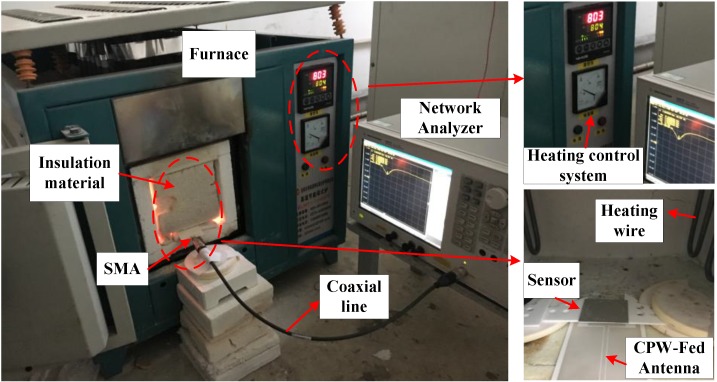
The image of the temperature test platform.

The image of the temperature measurement is shown as Figure 10. The rise curve of the furnace was set to increase 100 °C every 30 min, and to record the data at 50 °C, 100 °C, 150 °C, etc. At each temperature point where the data needed to be recorded, the furnace temperature was held constant for two minutes to ensure the accuracy of the test. In addition to the frequency-return loss S(1, 1) curves, the quality factor of the sensor at each temperature point was recorded manually.

## 5. Result and Analysis

**Figure 11:**
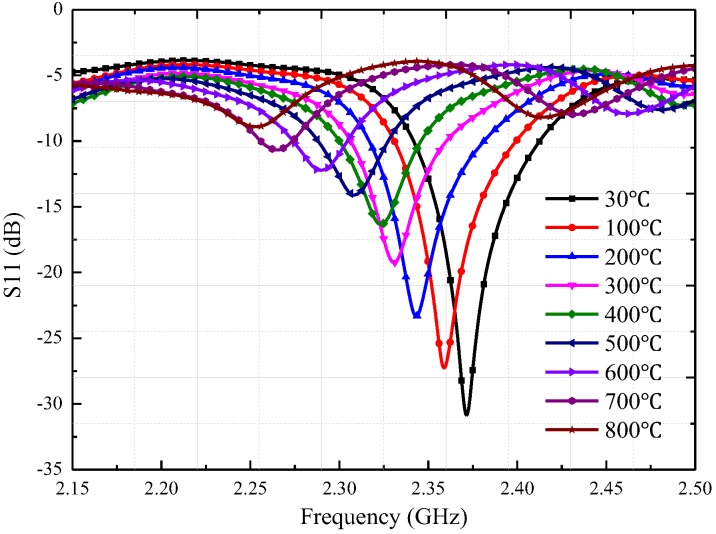
Temperature test at a sensing distance of 20 mm.

**Figure 12:**
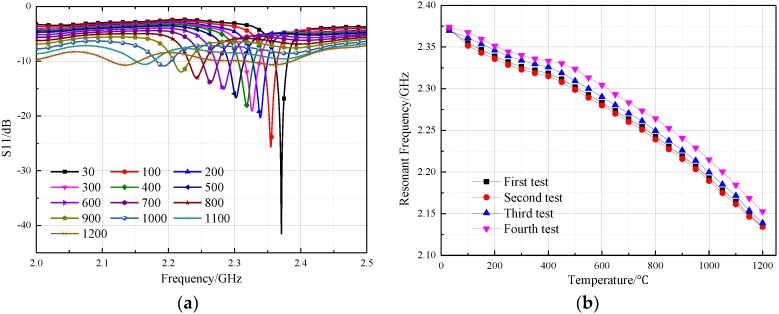
Temperature test result of the sensor (**a**) the result of the first test (**b**) repetitive test results.

During the first high temperature test, we adjusted the test distance between the sensor and CPW-fed square-slot antenna to 20 mm. The recorded return loss frequency curves were transmitted to the computer, the negative peak point represents the resonant frequency of the sensor at this temperature. As shown in Figure 11, the |S(1, 1)| rapidly attenuates during temperature increase when the sensing distance is 20 mm, and the peak value of the return loss frequency curves at 800 °C has become very small. Then, we adjusted the distance between the temperature sensor and CPW-fed square-slot antenna to five millimeters in order to obtain a much larger |S(1, 1)|, a higher quality factor and make the sensor signals tolerant to higher temperatures. At the sensing distance of five millimeters, we achieve the temperature test at 1200 °C, the return loss–frequency curves were recorded as shown in Figure 11a, and then the negative points of each curve were extracted. In this paper, four temperature tests (30 °C to 1200 °C) were conducted on the prepared sensor, as shown in Figure 12a.

**Figure 13:**
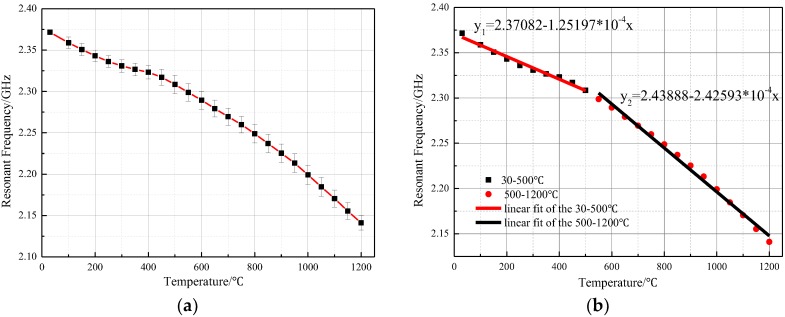
Average resonant frequency of the four tests (**a**) relationship between resonant frequency and temperature (**b**) Linear fitting curves.

As shown in Figure 12a, the resonant frequency of the sensor is 2.371 GHz at 30 °C, which deviates slightly from the simulation result. According to the dielectric perturbation theory of the substrate-integrated waveguide, the main reason for this deviation is that the dielectric constant of the substrate material was not exactly equal to 9.8 at 30 °C. The other reason is processing errors in the alumina ceramic substrate material. The resonant frequency of the sensor changed from 2.371 to 2.141 GHz when the temperature varied from 30 to 1200 °C as shown in Figure 8a. By extracting the negative peak of these curves, the relationship between the temperature and the sensor resonant frequency can be obtained. Additionally, the sensitivity of the temperature sensor presented in this paper can be expressed by Equation (4), and ∆*f_r_* represent the offset of the resonant frequency of the sensor, and ∆*T* represent the temperature change. Four temperature tests were conducted to verify the feasibility of the sensor, as shown in Figure 12b. For a more intuitive analysis of the repeated temperature test results, the average resonance frequency with error bars varying with the temperature is presented in Figure 13a.

(4) $$S=\frac{\Delta f_{r}}{\Delta T}$$

As shown in Figure 13a, the maximum standard deviation on the error bar curve is 11.4 MHz, appearing at 850 °C. Compared with the sensor in [8], the numerical variability between the four test results is smaller, which means that the temperature test error is smaller Then we came to a conclusion that the temperature sensor proposed in this paper has good test repeatability. Through the piecewise linear fitting (30 to 500 °C, and 500 to 1200 °C) it was found that the sensor sensitivity is 0.124 MHz/°C at the range of 30–500 °C, 0.243 MHz/°C at the range of 500–1200 °C, and the average sensitivity at the range of 30–1200 °C is 0.197 MHz. As shown in Equation (2), the resonant frequency of the sensor is not only affected by the relative dielectric constant of the material, but also by the relative position of the sidewall metal cylinders. The thermal expansion coefficient of the alumina substrate material increases with the increasing temperature, which was tested using the Unitherm^TM^ 1252 ultra-high temperature dilatometer. The results showed that the average thermal expansion coefficient of the 99 alumina ceramic is about 6.8 (1 × 10^−6^/K) in the range of 30–400 °C, while in the range of 500–1200 °C, it is around 9.5 (1 × 10^−6^/K). Thus, the increasing temperature causes the relative position of the sidewall metal cylinder to increase, which can explain why the test sensitivity of the sensor is greater in the range of 500–1200 °C.

During the test process, the sensor quality factor can be calculated and displayed in real time by the algorithm that comes with the network analyzer. The algorithm is based on the following Equation (5) to calculate the quality factor of the sensor, where *f*_0_ represents the center frequency of the sensor. Here the bandwidth *f*_−3*dB*_ is defined as the frequency difference between the two frequency points were selected where peak S_11_ lifted for three decibels. Then the quality factor of the sensor is shown in Table 3.

(5) $$Q=\frac{f_{0}}{\left|f_{-3dB}\right|}$$

As shown in Table 3, the temperature sensor quality factor of the in this paper is 770 at 100 °C and decrease to 35 at 1000 °C, because the dielectric loss of the substrate material increases with the change of temperature. In addition, the sensor quality at the 30 °C is significantly greater than the quality factor of the simulation result, which is mainly caused by the difference of the relative position between the sensor and antenna. 

Through the analysis, it can be concluded that the sensor proposed here has high sensitivity, low profile, high quality factor, and a wide temperature sensing range. Table 4 shows the visualized parameters of the SICW temperature sensor we fabricated and other kinds of wireless temperature sensors available in literature.

As shown in Table 4, the SICW temperature sensor proposed here has great test sensitivity compared with the slot radiation patch sensor in [27], and the SIW structure has great quality factor than the sensor in [27]. Compared with the LC-based sensor, the SIW structure sensor can work at a microwave frequency band with a small dimension, while the LC sensor works at a lower frequency band in the 50–500 MHz range, and the SIW sensor is more sensitivity than the LC temperature sensors in [6,7]. Compare with the dielectric resonance temperature sensor in [8], the thickness of the sensor in this paper is lower, and the installation on the surface of the metal blade can be realized more easily. Additionally, the sensor in this article has a great sensitivity at the high temperature (above 500 °C) than the sensor in [8], the quality factor at high temperature of the sensor in this article is higher than the sensor in [8]. Besides this, compare the sensor in this paper with these conventional microwave temperature sensors in [26,27,28]; this SICW sensor has great sensing range, and achieves the temperature test at 1200 °C, because it was fabricated with Pt. For the sensing distance, here the sensor test in this paper belongs to the near-field coupling for the using of slot antenna, and longer sensing distances will be explored in future research.

## 6. Conclusions

The temperature sensor presented in this paper was based on the substrate integrated waveguide structure, and the 99 Alumina ceramic was chosen as the substrate material. Platinum paste was used to form the upper and lower metal plates of the SIW structure, and to fill up the sidewall cylindrical vias. The high temperature test platform consisted of a furnace, a coaxial line, a CPW fed-slot antenna and the network analyzer. Through multiple experimental tests, it was found that the resonant frequency of the sensor changed from 2.371 to 2.141 GHz when the temperature varied from 30 to 1200 °C, leading to an average sensitivity of 0.197 MHz/°C. The quality factor of the sensor was about 35.028 at 1000 °C. The performance of the sensor at 1200 °C showed that this type of sensor structure has the potential to be tested in harsh environments with high temperatures.

The sensor presented in this paper was not tested above 1200 °C because the CPW-fed slot antenna broke down at about 1240 °C during the first test. When the temperature was above 1200 °C, the temperature difference between the parts of the antenna inside and outside of the furnace reached the limit value, which produced a thermal stress sufficient to cause the antenna to break down. In future work, we will focus on optimizing the interrogation antenna structure to support measurements at higher temperatures. In addition, we will consider how to optimize the size and thickness of this type of sensor so that it can be installed on engine blades.

## Figures and Tables

**Table 1:** The initial dimensions parameter of the substrate integrated circular waveguide (SICW) (mm).

*L*	*W*	*R*	*D*	*b*	*h*
35	35	15.5	1	2.44	1

**Table 2:** The resonant frequency and the quality factor of the sensor under the different thickness of the substrate material.

The Thickness of the SIW (mm)	Resonant Frequency (GHz)	Quality Factor
0.7	2.39427	498.121
0.8	2.3948	563.360
0.9	2.39488	628.554
1.0	2.39529	690.88
1.1	2.39556	753.529
1.2	2.39545	815.395
1.3	2.39539	875.384

**Table 3:** The quality factor of the sensor at different temperature.

Temperature (°C)	Q	Temperature (°C)	Q
100	770.64	600	148.58
200	374.12	700	113.20
300	314.18	800	91.331
400	253.11	900	66.990
500	206.4	1000	35.028

**Table 4:** Parameters of different temperature sensors.

Sensor Type	Profile	Sensitivity	Temperature Sensing Range	Sensing Distance	Working Frequency
SICW sensor	35 mm × 35 mm × 1 mm	197 KHz/°C	25–1200 °C	About 60 mm	Around 2.27 GHz
Slot radiation patch sensor in [27]	40 mm × 40 mm × 1 mm	101.94 KHz/°C	25–800 °C	Maximum 14 mm	Around 2.31 GHz
Resonator based microwave sensor in [28]	22 mm × 22 mm × 1.5 mm	0.24 MHz/°C	50–400 °C	Maximum 30 mm	Around 2.28 GHz
Inductive capacitive (LC) resonance sensor in [7]	36 mm × 36 mm × 0.68 mm	Maximum 16.67 KHz/°C	25–700 °C	10 mm	Around 33.5 MHz
Surface acoustic wave (SAW) based sensor in [29]	20 (dia)	/	Maximum +250 °C	Above 10 cm	Around 2.44 GHz
Dielectric resonance temperature sensor in [8]	29 mm × 29 mm × 5 mm	194 KHz/°C	27–800 °C	About 10 mm	Around 2.44 GHz
